# A Combinatorial Amino Acid Code for RNA Recognition by Pentatricopeptide Repeat Proteins

**DOI:** 10.1371/journal.pgen.1002910

**Published:** 2012-08-16

**Authors:** Alice Barkan, Margarita Rojas, Sota Fujii, Aaron Yap, Yee Seng Chong, Charles S. Bond, Ian Small

**Affiliations:** 1 Institute of Molecular Biology, University of Oregon, Eugene, Oregon, United States of America; 2 Centre of Excellence in Computational Systems Biology, The University of Western Australia, Crawley, Western Australia, Australia; 3 Australian Research Council Centre of Excellence in Plant Energy Biology, The University of Western Australia, Crawley, Western Australia, Australia; 4 School of Chemistry and Biochemistry, The University of Western Australia, Crawley, Western Australia, Australia; University of Minnesota, United States of America

## Abstract

The pentatricopeptide repeat (PPR) is a helical repeat motif found in an exceptionally large family of RNA–binding proteins that functions in mitochondrial and chloroplast gene expression. PPR proteins harbor between 2 and 30 repeats and typically bind single-stranded RNA in a sequence-specific fashion. However, the basis for sequence-specific RNA recognition by PPR tracts has been unknown. We used computational methods to infer a code for nucleotide recognition involving two amino acids in each repeat, and we validated this model by recoding a PPR protein to bind novel RNA sequences *in vitro*. Our results show that PPR tracts bind RNA via a modular recognition mechanism that differs from previously described RNA–protein recognition modes and that underpins a natural library of specific protein/RNA partners of unprecedented size and diversity. These findings provide a significant step toward the prediction of native binding sites of the enormous number of PPR proteins found in nature. Furthermore, the extraordinary evolutionary plasticity of the PPR family suggests that the PPR scaffold will be particularly amenable to redesign for new sequence specificities and functions.

## Author Summary

RNA binding proteins dictate RNA fate and function by modulating RNA processing, localization, translation, and stability. The consequences of RNA/protein interactions are determined, in part, by the position at which the protein binds the RNA. However, it is impossible to predict the target sites of most RNA binding proteins or to design them to bind chosen RNA sequences. In contrast, we show that the pentatricopeptide repeat (PPR) protein family holds exceptional promise for the rational design of specified RNA–binding properties. PPR proteins harbor tandem arrays of a repeating structural unit that form a surface for binding single-stranded RNA. We show that PPR tracts bind specific RNA nucleotides via the combinatorial action of two amino acids in each repeat. This mechanism mimics the simplicity and predictability of the Watson-Crick pairing between nucleic acid strands, but at a protein/RNA interface. Our findings will facilitate the prediction of binding sites for the large number of PPR proteins found in nature. Additionally, our demonstration that a PPR tract can be engineered to bind specified RNA sequences implies that PPR proteins can be designed to bind desired RNA targets for applications in biotechnology, medicine, and basic research.

## Introduction

Much of modern biology deals with understanding and predicting macromolecular interactions. The biotechnological possibilities inherent in being able to predict, design and manipulate macromolecular interactions are immense. The well-understood Watson-Crick pairing between nucleic acid strands facilitates the design of nucleic acids that can interact with specific DNA or RNA sequences, and this ability underlies a huge swathe of modern research and biotechnology. Given the greater functional potentialities of proteins compared to nucleic acids and the ability to target proteins to different intracellular compartments, new opportunities would emerge from the ability to design proteins to bind specific RNA or DNA sequences. Unfortunately, most protein-nucleic acid interactions are idiosyncratic, and lack the predictability necessary to engineer specific interactions. Recently, a great deal of excitement has accompanied the characterization of Transcription-Activator-Like Effectors (TALEs), a set of modular repeat proteins that bind via a predictable code to specific double-stranded DNA sequences [1], [2]. TALEs belong to the alpha-solenoid superfamily comprising proteins that consist of degenerate repeats of 30–40 amino acids, each of which forms two or three alpha-helices. This superfamily includes only one well characterized member that binds RNA: the Puf domain family. Puf domains consist of eight tandem repeats of a triple-helix motif that bind 8–9 nucleotide sites (reviewed in [3]). The residues within each motif that dictate sequence specificity have been identified, and experiments to manipulate binding specificity and protein function by exploiting this modular recognition have been successful [3], [4], [5].

This study focuses on a second class of helical repeat motif that binds RNA, the pentatricopeptide repeat (PPR). PPR proteins harbor degenerate ∼35 amino acid repeats that are related to tetratricopeptide (TPR) motifs [6]. PPR proteins localize primarily to mitochondria and chloroplasts where they influence various aspects of RNA metabolism [7]. Many PPR proteins are essential for photosynthesis or respiration, and mutations in PPR-encoding genes are associated with genetic diseases in humans (e.g. [8]). Although less widely known than Pufs and TALEs, PPR proteins are much more prevalent in nature. Protist, fungal and metazoan genomes encode roughly 5–50 PPR proteins, but the family has expanded to >400 members in plants (reviewed in [9]). The products of evolution illustrate the apparent ease with which PPR tracts can be modified to bind diverse sequences and mediate diverse functions: PPR proteins harbor between 2 and ∼30 repeats and they influence the processing, editing, splicing, stability or translation of specific organellar RNAs [7]. The remarkable evolutionary plasticity of PPR proteins is highlighted by their natural exploitation to silence rapidly evolving mitochondrial open reading frames that confer cytoplasmic male sterility in plants [10].

Results presented here demonstrate that PPR tracts bind RNA via a modular mechanism that conceptually resembles Puf-RNA recognition. However, the details of nucleotide recognition by PPR motifs differ from those for Puf repeats, revealing a diversity of independently evolved RNA recognition modes by alpha solenoid repeats. These insights provide a significant step toward the prediction of binding sites and functions for the large number of PPR proteins found in nature. Additionally, the evolutionary malleability of the PPR family implies that PPR binding specificities can be engineered to match a wide variety of desired targets.

## Results

To develop models for sequence-specific RNA recognition by PPR tracts, we began with a focus on the maize protein PPR10, whose binding sites and mechanisms are particularly well understood [11], [12]. PPR10 consists of 19 PPR motifs and little else. PPR10 localizes to chloroplasts, and binds two different RNAs via *cis*-elements with considerable sequence similarity. PPR10 serves to position processed mRNA termini and stabilize adjacent RNA segments *in vivo* by blocking exoribonucleases intruding from either direction.

### PPR10 Binds RNA as a Monomer

**Figure 1:**
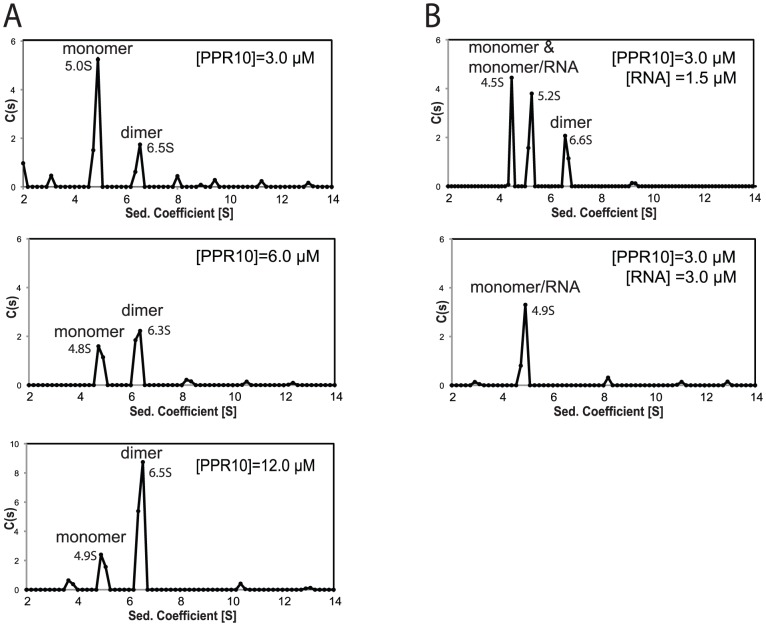
Sedimentation Velocity Analytical Ultracentrifugation of rPPR10 and rPPR10/RNA Complexes. (A) SV-AUC analysis of rPPR10 at 3, 6, and 12 µM. (B) SV-AUC analysis of rPPR10 (3 µM) in the presence of its 17-nt minimal RNA ligand (1.5 µM or 3 µM). The assignment of the two species at ∼5S in the top panel as either PPR10 monomer or PPR10/RNA is ambiguous, as variation in apparent S value can result when multiple species of similar abundance are in equilibrium. The root-mean-squared-deviations ranged between .007 and .013. The trace species at low S values may result from contaminating MBP and TEV protease, whereas those of larger size may represent higher order PPR10 oligomers.

Recombinant PPR10 (rPPR10) elutes from a gel filtration column at a position corresponding to a globular homodimer [11], as does HCF152, which likewise consists almost entirely of PPR motifs [13]. Models for PPR-RNA interaction would need to incorporate homodimerization, should this be physiologically relevant. To clarify this point, we analyzed rPPR10 by sedimentation velocity analytical ultracentrifugation (SV-AUC). rPPR10 was found predominantly in two forms whose ratio changed in a concentration-dependent fashion (Figure 1A). At 3 µM, the major species sedimented at ∼5 S and had an estimated molecular weight of 84.9 kDa, close to rPPR10's monomeric molecular weight of 82.6 kDa. A two-fold increase in rPPR10 concentration shifted the distribution toward a larger species (∼6.5 S), which predominated when protein concentration was further increased to 12 µM. These results strongly suggest the ∼5 S and 6.5 S species to be monomers and dimers, respectively. Thus, rPPR10 can dimerize, but only at very high concentrations.

To determine which form of PPR10 binds RNA, rPPR10 was analyzed by SV-AUC in the presence of its 17-nt minimal RNA ligand. This RNA is small in comparison with rPPR10 (5 kDa *versus* 84 kDa) and does not contribute significant signal with the interference optical system used for these experiments. With rPPR10 at 3 µM and RNA at half that concentration, PPR10 monomers partitioned into two species of similar abundance with an S value near 5 S (Figure 1B). The concentration, sedimentation rate, and RNA-dependence of the second ∼5S species strongly suggest it to be a PPR10 monomer bound to RNA. The pair of species near 5S collapsed into a single ∼5 S species when the RNA concentration was increased to be equimolar with PPR10 (3 µM). As this concentration is much higher than the K_d_ for the PPR10-RNA interaction (<1 nM) [12], it is predicted that essentially all of the protein was bound to RNA, assuming a 1∶1 stoichiometry. Taken together, these results provide strong evidence that PPR10 binds RNA in its monomeric form, and that each PPR10 monomer binds one RNA molecule. Under conditions of saturating RNA, PPR10 dimers were not detected. Thus, RNA binding appears to preclude protein dimerization, suggesting that PPR10's RNA binding and dimerization surfaces overlap.

### Modeling the Polarity and Register of a PPR10-RNA Complex Suggested an Amino Acid Code for RNA Recognition

**Figure 2:**
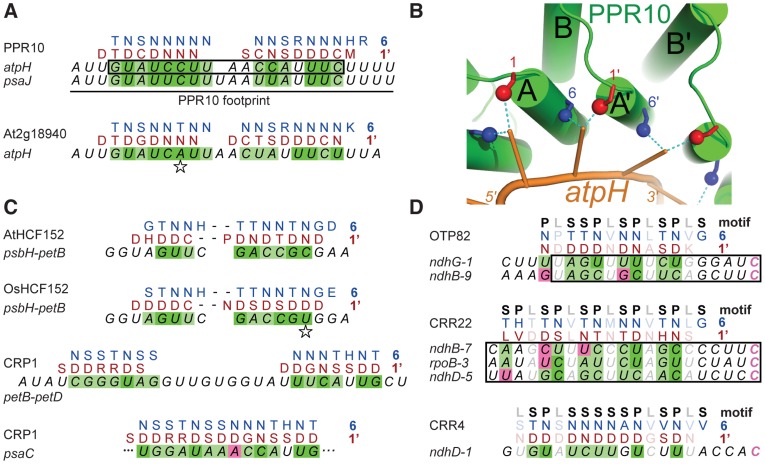
Alignments between PPR Proteins and Cognate Binding Sites. (A) Statistically optimal alignments between amino acids at positions 6 (blue) and 1′ (red) in PPR10's PPR motifs and its RNA ligands (italics). PPR10's *in vivo* footprints are shown at top; the box marks the minimal binding site defined *in vitro*. Dark green shading indicates experimentally validated matches (Figure 5). Light green shading indicates significant correlation between position 6 and the purine/pyrimidine class of the matched nucleotide (Table S3). Magenta shading indicates significant anti-correlation between position 6 and the purine/pyrimidine class of the matched nucleotide (Table S3). Compensatory changes in orthologous protein/RNA pairs are indicated with a star. The PPR motifs are ordered from N to C terminus in the protein, and nucleotides are ordered from 5′ to 3′ in the RNA. The same schemes apply to panels (C) and (D). (B) Structural model illustrating physical plausibility of the cooperation between amino acids at positions 6 and 1′ in nucleotide specification. The model of the PPR10-*atpH* RNA complex was produced using distance geometry methods as previously described [10]. RNA bases were constrained to be within 3 Å of residues 6 and 1′ of helices A and A′ of adjacent motifs. Each PPR motif consists of one “A” and one “B” helix, as marked. (C) Alignments between amino acids at positions 6 and 1′ in PPR motifs of HCF152 and CRP1 and their RNA ligands. The *psbH-petB* sequence is HCF152's *in vivo* footprint [17], within which HCF152 binds *in vitro* [16]. The *petB-petD* sequence is a CRP1-dependent *in vivo* footprint [16]. The *psaC* sequence maps within the 70-nt region that most strongly coimmunoprecipitates with CRP1 [19]. (D) Alignments between amino acids at positions 6 and 1′ in PPR motifs of the RNA editing factors OTP82, CRR22 and CRR4 and their RNA targets [14], [29]. Minimal binding sites determined *in vitro* are boxed. The edited C (magenta) is the last nucleotide in each case. The type of PPR motif, either P, L or S, is indicated above. Only matches involving P or S motifs are shaded, as L motifs cannot be accommodated within the code developed here.

The minimal PPR10 binding site in the *atpH* 5′-UTR spans 17-nt and PPR10 leaves a ribonuclease-resistant footprint spanning ∼24 nucleotides [12] (Figure 2A). To identify specificity determining amino acids, we sought correlations between the amino acid residues at each position of PPR10's PPR motifs and the bases within its footprint. We modeled the RNA in parallel to the protein (i.e. 5′-end aligned with N-terminus) due to the organization of PPR proteins that specify sites of RNA editing: such proteins have an N-terminal PPR tract and a C-terminal domain that is required for editing, and they bind *cis*-elements that are 5′ of the edited sites (reviewed in [7]). We further assumed that all motifs would contact an RNA base, but not necessarily contiguously. These assumptions are based on the similarity between the number of repeats and the number of nucleotides in well-characterized PPR/RNA pairs [12], [14], and by a length polymorphism in the middle of PPR10's two binding sites (Figure 2A).

Given these constraints, there are 420 possible arrangements of PPR10's PPR motifs in contact with its RNA footprint (see Materials and Methods). One of these arrangements stood out because it showed strong correlations between the RNA base and the amino acids found at positions 1 and 6 (Table S1 and Figure 2A), which were suggested to be specificity-determining positions based on their patterns of evolutionary selection [10]. The alignment to amino acid 6 is offset by one nucleotide from the alignment to amino acid 1, such that the base that correlates with position 6 of PPR motif *n* also correlates with position 1 of the *n+1* motif; hereafter we shall refer to this position as 1′, to distinguish it from position 1 in motif *n*. This offset is physically plausible (Figure 2B), and it is supported by an *in vitro* analysis of a pair of PPR motifs [15]. The optimal alignment contains a gap that breaks the protein-RNA duplex into two segments. The gap corresponds with the position of a single nucleotide insertion in PPR10's *psaJ* binding site (Figure 2A), providing evidence for relaxed selection in this region of the binding site. This alignment highlights the following correlations: every N_6_ aligns with a pyrimidine, each purine corresponds to S_6_ or T_6_, and every D_1′_ aligns with a U. These correlations are maintained by covariation when one considers the orthologous protein and binding site in Arabidopsis (Figure 2A).

These correlations were extended by analysis of the PPR protein HCF152 [13], which binds to sequences within its 17-nt footprint in the chloroplast *psbH-petB* intergenic region [16], [17]. When HCF152's 13 PPR motifs were compared with this sequence, the optimal alignment spanned 12 nucleotides and preserved the correlations observed for PPR10 (Figure 2C). Furthermore, this alignment is maintained through covariation in rice (Figure 2C). The maize protein CRP1 further strengthens these correlations. CRP1 leaves a ∼30-nt footprint in the chloroplast *petB-petD* intergenic region [16], [18]. CRP1's 14 PPR motifs can be aligned within this footprint in a manner that retains the correlations noted above (Figure 2C). Similar to the PPR10 alignments, the CRP1 alignment involves ∼7 contiguous matches at each end, with “unpaired” nucleotides in the central region. Notably, the PPR10, HCF152, and CRP1 alignments are all placed very similarly within their RNAse-resistant footprints, as is to be expected given that each protein blocks access by the same exonucleases *in vivo*. Finally, an alignment that follows the same rules can be made between CRP1 and a sequence in the *psaC* 5′-UTR that maps within the 70-nt segment that is most strongly enriched in CRP1 coimmunoprecipitations [19] (Figure 2C).

PPR proteins can be separated into two classes, denoted P and PLS. PPR10, HCF152, and CRP1 are examples of P-class proteins, which contain tandem arrays of 35 amino acid PPR motifs. Members of this class have been implicated in RNA stabilization, processing, splicing, and translation. PLS-class proteins contain alternating canonical ‘P’ motifs and variant ‘long’ and ‘short’ PPR motifs [20], and typically function in RNA editing. PPR editing factors can be aligned to sequences upstream of the edited nucleotide such that the amino acids at position 6 of the ‘P’ motifs and the amino acids at position 1′ of the following ‘L’ motif correlate with the matched nucleotide in a similar manner to that found for the P-class proteins (Figure 2D). Importantly, the editing factors can all be aligned such that their C-terminal motif is at the same distance from the edited cytidine residue. This not only explains how the target C is defined, it allows the motif-nucleotide correlations in the editing factors to be evaluated without using them to make the alignment. Correlations between the aligned base and the amino acids at positions 6 and 1′ are highly significant across all alignments for both ‘P’ and ‘S’ motifs (Table S2). Apart from these two positions, only the amino acid at 4′ is also significantly correlated with the aligned nucleotide.

**Figure 3:**
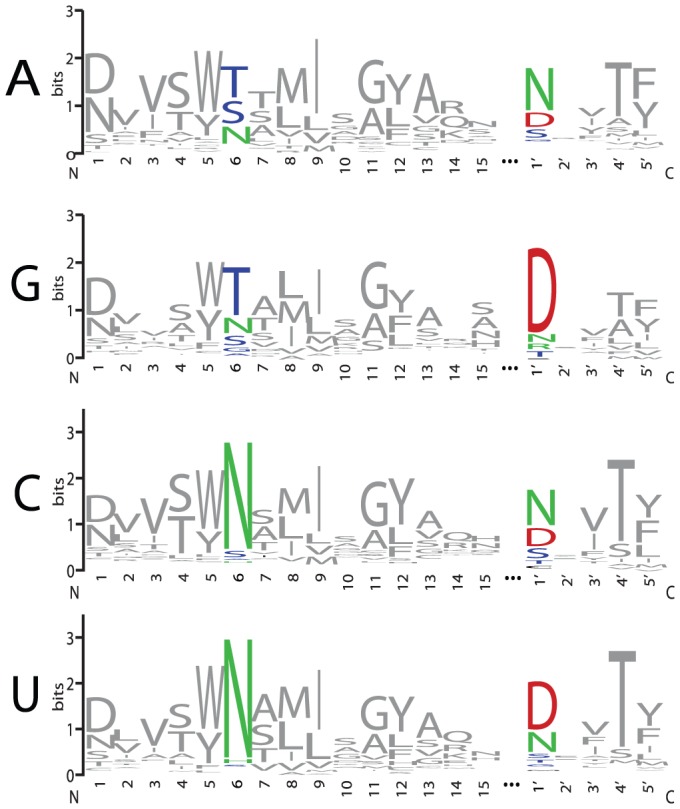
Amino Acid Representation at Each Position of PPR Motifs that Align with A, G, C, or U Bases. Motif pairs from PPR10, HCF152, CRP1 and 37 RNA editing factors flanking the indicated nucleotide were used to construct sequence logos [30]. Each logo shows the first fifteen positions of the P-type motif containing position 6, a gap, and then the first 5 positions of the following motif. 74, 48, 96 and 126 motif pairs were used to generate the A, G, C and U logos, respectively. The alignments used to generate the logos are shown in Figure S1.

**Figure 4:**
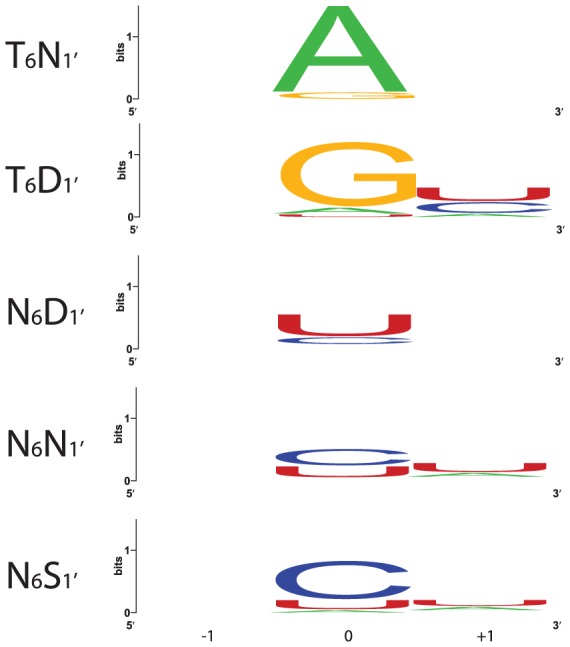
Nucleotides That Align with the Most Frequent Combinations of Amino Acids at Positions 6 and 1′. Nucleotides aligned with each 6/1′ combination in the alignments in Figure S1 were used to construct sequence logos [30]. Only P motifs were used in this analysis. Each logo shows the aligned nucleotide (0) and the preceding (−1) and succeeding (+1) nucleotides. 25, 23, 102, 86 and 16 alignments were used to generate the T_6_N_1′_, T_6_D_1′_, N_6_D_1′_, N_6_N_1′_ and N_6_S_1′_ logos, respectively.

Sequence logos constructed from PPR motif pairs aligned with either A, G, C, or U are shown in Figure 3 and Figure 4. From these alignments, a set of rules can be derived that seem likely to represent a combinatorial amino acid code for nucleotide recognition by PPR motifs: T_6_D_1′_ = G; T/S_6_N_1′_ = A; N_6_D_1′_ = U; N_6_N/S_1′_ = C. The diversity of amino acid combinations at these positions implies that the code may be degenerate (Table S3). However, the above-mentioned amino acid combinations are the most commonly observed, and together represent 64% of all canonical PPR motif pairs in Arabidopsis and rice (Figure S2).

### Confirmation of a Code by Recoding PPR10 to Bind New RNA Sequences

**Figure 5:**
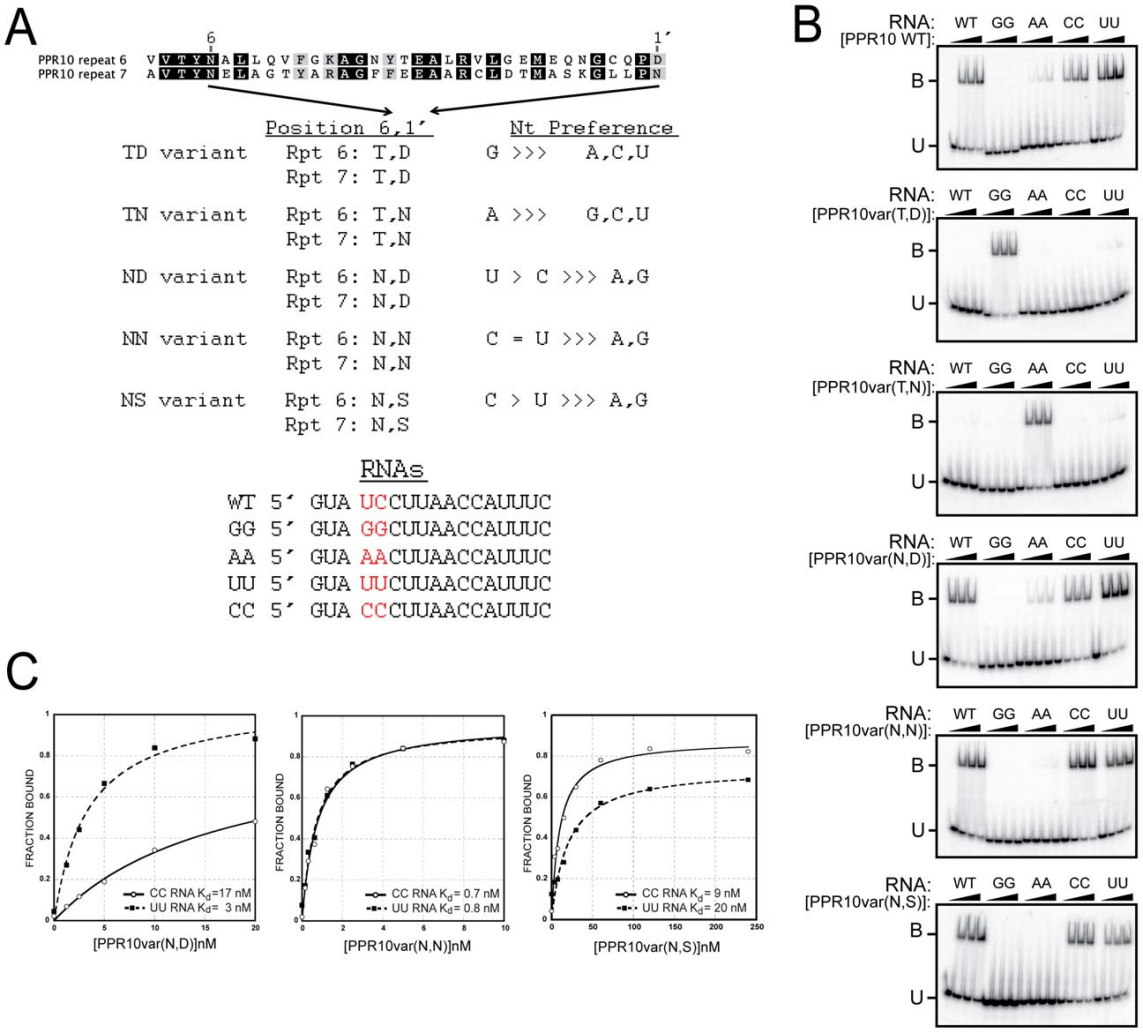
Gel Mobility Shift Assays Validating Amino Acid Codes for Specifying PPR Binding to A, G, C, or U. (A) Summary of rPPR10 variants. The same amino acids at positions 6 and 1′ were introduced into the sixth and seventh PPR motifs in PPR10, whose wild-type sequences are shown above. The RNAs used for binding assays are shown below. (B) Gel mobility shift assays with the wild-type RNA, or variants with nucleotides four and five substituted with either GG, AA, UU, or CC. (C) Binding curves of the NN, ND, and NS PPR10 variants with the UU and CC substituted RNAs.

To test whether the correlations between amino acid identities at PPR positions 6 and 1′ and the associated nucleotide reflect a recognition code, we generated a set of PPR10 variants in which residues (6, 1′) in a pair of adjacent repeats (motifs six and seven) were modified to either T_6_D_1′_, T_6_N_1′_, N_6_D_1′_, N_6_N_1′_, or N_6_S_1′_ (Figure 5A). Our model aligns PPR10 repeats 6 and 7 with U and C nucleotides, respectively. PPR10 does not bind significantly to RNA in which these nucleotides are substituted with either AA or GG (Figure 5B). A PPR10 variant in which motifs 6 and 7 were modified to (T,D) did not bind to the wild-type RNA, but bound with high affinity to RNA with the GG substitution. Likewise, the variant in which these motifs were modified to (T,N) did not bind to wild-type RNA, but bound with high affinity to RNA with the AA substitution. Neither variant bound significantly to any of the other substituted RNAs. These results confirmed the proposed polarity and register of the PPR10/RNA complex, and show that (T,D) and (T,N) at positions (6, 1′) are highly specific for binding G and A, respectively.

The (N,D), (N,N), and (N,S) combinations at (6, 1′) correlate with recognition of pyrimidines (Figure 4 and Table S3). As predicted, PPR10 variants with these amino acid combinations strongly favored binding to pyrimidine-substituted RNAs (Figure 5B). The (N,D) variant bound the U and C substituted RNAs with K_d_s of ∼3 nM and 17 nM, respectively, indicating a clear preference for U over C (Figure 5C). Conversely, the (N,S) variant favored C over U, albeit only slightly (K_d_s of 9 nM and 20 nM for the C and U substituted RNAs, respectively). The (N,N) variant is less discriminating, binding the U and C substituted RNAs with similar affinities (Figure 5C).

## Discussion

Results presented here provide strong evidence that PPR tracts bind RNA in a parallel orientation via a modular recognition mechanism, with nucleotide specificity relying primarily on the amino acid identities at positions 6 and 1′ in each repeat. Modification of amino acids at these positions in the context of two adjacent PPR motifs was sufficient to change the nucleotide preference, suggesting that other amino acid positions make no more than a small contribution to nucleotide specificity. Position 4′ correlates weakly with the aligned nucleotide, but threonine is preferred at 4′ for all four nucleotides (Figure 3) and we have not investigated the effect of any other amino acid at this position. Although similar in concept to Puf/RNA recognition, PPR/RNA complexes have the opposite polarity and involve distinct amino acid combinations. The polarity and code we demonstrate for PPR/RNA interactions differ from those proposed by Kobayashi et al [15], who concluded that the PPR protein HCF152 binds anti-parallel to an A-rich RNA sequence. This model was based on a shallow HCF152 SELEX dataset, from which similarities were sought to a presumed HCF152 binding site that was recently shown not to bind HCF152 with high affinity [16].

Our results define a combinatorial two-amino acid code that can specify binding of a PPR motif to either A, G, U>C, C>U, or U = C. With this knowledge, the engineering of PPR tracts to bind a wide variety of RNA sequences is within reach. However, prediction of the natural binding sites of PPR proteins, and prediction of off-target binding by engineered PPR proteins remains challenging for two reasons. First, the natural diversity of amino acid identities at positions 6 and 1′ implies a degenerate code, and less than two-thirds of naturally occurring combinations can currently be interpreted. Second, an understanding of the energetic parameters required to establish a physiologically meaningful PPR/RNA interaction and the energetic costs of mismatches at various positions along a PPR/RNA duplex will be required to accurately predict potential binding sites. The prediction of microRNA targets is similar in concept and provides a glimpse into the challenge to come: despite the simplicity of RNA base pairing rules, the parameters that dictate microRNA targets are still being worked out [21].

Prediction of binding sites is further complicated by the fact that gaps in a PPR/RNA duplex can be tolerated in some contexts, as exemplified by PPR10's natural targets (Figure 2A). Indeed, the optimal alignments of the P-class PPR proteins HCF152 and CRP1 also contain a gap, with the predicted protein/RNA duplex containing non-contiguous segments of either RNA (PPR10 and CRP1) or protein (HCF152). These gaps break the protein-RNA duplex into two segments in a manner that resembles Puf-RNA duplexes, which require contiguous protein-RNA matches at each end but can accommodate various flipped base conformations in the central region [22]. Our findings imply considerable flexibility in the length of the “looped out” RNA between contiguous PPR-RNA segments. These RNA loops may be analogous to internal loops in RNA duplexes, which adopt diverse architectures due to the great flexibility of the RNA backbone and to the wealth of opportunities for non-canonical base-base interactions (reviewed in [23], [24]).

Our alignments of P-class PPR proteins to their cognate RNAs include contiguous duplexes consisting of no more than nine motifs and eight nucleotides. This is reminiscent of the binding of 8–9 nucleotides by the eight repeats in Puf proteins (reviewed in [25]). The number of contiguous interactions between helical repeats and RNA bases may be constrained by the minimum distance between parallel alpha helices. The minimum theoretical helix-helix distance is *c.* 9.5 Å [26], which is approached by the helix-helix distance in Puf motifs [27]. In contrast, adjacent nucleotides in Puf:RNA complexes are 7 Å apart, close to the maximally extended conformation, and resulting in a distance mismatch that is only partially accommodated by curvature of the RNA-binding surface. A similar constraint may limit the maximum number of contiguous RNA bases bound by tandem PPR motifs. There is no evidence for gaps in the alignments between PLS-class editing factors and their RNA targets. However, the representation of amino acids at position 6 differs between P and S *versus* L-type PPR motifs. Thus, we suspect that L motifs do not bind nucleotide bases, allowing a ‘mini-gap’ every third nucleotide that may relax the structural constraints.

The well-defined code for RNA recognition by Puf domains provides a means to engineer proteins to bind specified RNA sequences. Results presented here imply that PPR tracts could be exploited for similar purposes. In fact, PPR tracts may well offer functionalities beyond those achievable with engineered Puf domains due to their more flexible architecture. Unlike Puf domains, whose 8-repeat organization is conserved throughout the eucaryotes, natural PPR proteins have between 2 and ∼30 repeats and rapidly evolve to bind new RNA sequences and fulfill new functions (reviewed in [9]). The unusually long surface for RNA interaction that is presented by long PPR tracts has the potential to sequester an extended RNA segment, which can impact RNA function in novel ways [12]. PPR proteins play essential roles in all eucaryotes by enabling the expression of specific mitochondrial and chloroplast genes. Even for well-studied PPR proteins such as human LRPPRC (e.g. [8]), the exact binding sites still await discovery. The results and approaches described here offer the potential to eliminate this bottleneck by permitting candidate sites to be postulated from simple sequence analysis, providing information that will have broad application in the medical and agricultural sciences.

## Materials and Methods

### Expression of rPPR10

rPPR10 and its variants were expressed in *E. coli* and purified as in [11]. In brief, mature PPR10 (lacking the plastid targeting peptide) was expressed as a fusion to maltose binding protein (MBP), purified by amylose affinity chromatography, separated from MBP by cleavage with TEV protease, and further purified by gel filtration chromatography in 250 mM NaCl, 50 mM Tris-HCl pH 7.5, 5 mM ß-mercaptoethanol. The elution peak was diluted in the same buffer for AUC, or dialyzed against 400 mM NaCl, 50 mM Tris-HCl pH 7.5, 5 mM ß-mercaptoethanol, 50% glycerol prior to use in RNA binding assays.

PPR10 variants were obtained by PCR-mutagenesis using the following primers (lower case indicates mutations): TD Variant: 5′ GGTCTGTTGCCAgACGCATTCACG; 5′ CGTGAATGCGTcTGGCAACAGACC; 5′ GCTGTGACGTACAcCGAGCTCGCCGGAACG ; 5′ CGTTCCGGCGAGCTCGgTGTACGTCACAGC ; 5′ CACCTGGAGCAACGCGgTGTACGTGACGACGCAC. TN Variant: 5′ CGTGAATGCGTtTGGCAACAGACCC; 5′ GGGTCTGTTGCCAaACGCATTCACG ; 5′ GAACGGCTGCCAGCCAaAcGCTGTGACGTAC ; 5′ CGgTGTACGTCACAGCgTtTGGCTGGCAGCCG. NN Variant: 5′ GGAGCAGAACGGCTGCCAGCCAaacGCTGTGACG; 5′ CGTCACAGCgttTGGCTGGCAGCCGTTCTGCTCC. ND Variant: 5′ GGTCTGTTGCCAgACGCATTCACG; 5′ CGTGAATGCGTcTGGCAACAGACC. NS Variant: 5′ GCTGCCAGCCAagcGCTGTGACG; 5′ CGTCACAGCgctTGGCTGGCAGC;5′ GTCTGTTGCCAagcGCATTCACGTACAACACC; 5′ GGTGTTGTACGTGAATGCgctTGGCAACAGAC

### Analytical Ultracentrifugation

SV-AUC was performed in a Beckman Optima XL-I ultracentrifuge with a Beckman An60Ti rotor. 400 µl of sample and 410 µl of reference buffer were analyzed in a 1.2 cm double-sector standard AUC cell. Experiments were run at 20°C at 50,000 rpm and monitored with an interference optical system. Data were collected at 3 min intervals for 8 hrs, and analyzed with SedFit [28], using a partial specific volume for rPPR10 of 0.73543 calculated from its amino acid composition. The residuals in all experiments were randomly distributed, and 95% of the residuals had a value <10% of the signal.

### Statistical Analysis of PPR/RNA Alignments

The alignment of PPR10 to its *atpH* binding site was generated *de novo* as follows. Thirty-five 17-mers were constructed, each corresponding to the amino acids at a specific position within the 17 sequential PPR motifs in PPR10's interior. Terminal PPR motifs were excluded, as they have distinct properties that may adapt them to their terminal position. These 17 motifs can be arranged in 420 different ways on the 24-nucleotides that are protected by PPR10, assuming that all the motifs contact the RNA sequentially but not necessarily contiguously, and permitting gaps of any length at any position. The number of arrangements is doubled if both polarities of the protein on the RNA are considered. For each of the 840 arrangements, contingency tables were constructed for each of the 35 17-mers, scoring the number of co-occurrences of each possible amino acid/nucleotide pair (i.e. a total of 29400 20×4 tables). Fisher's Exact Test was used to test for independence of amino acid and nucleotides classes, as implemented in R version 2.14.2 by fisher.test. The tables were ranked by p-value. The top ranked alignment (1/29400) was for position 1. The best alignment for position 6 was also retained (ranked 71/29400). No other highly ranked alignments were physically compatible with the motif arrangement required for the alignment shown in Figure 2A (i.e. contained a gap of the same length in the same place). The Figure 2A alignments are empirically supported by the boundaries of the PPR10 footprint and minimal binding site, by covariations among PPR10 orthologs and their binding sites, by natural variation in the central region of PPR10's two native binding sites, and by binding affinities of PPR10 for variant *atpH* sites with various insertions and point mutations [12].

#### Gel mobility shift assays

Gel mobility shift assays and K_d_ calculations were performed as described [12], using radiolabeled synthetic RNAs at 15 pM and protein at 0, 5, 10, and 20 nM, unless otherwise indicated.

## Supporting Information

Figure S1 Alignments of PPR editing factors to their target sites. For each factor, the name of the protein and its editing site are listed, then successively the types of PPR motif, the amino acids at position 6, the amino acids at position 1′, an indication of the degree to which these amino acids ‘match’ the RNA using the code developed in this work, and lastly the RNA sequence (in lower case). ‘:’ and ‘.’ indicate experimentally validated (see Figure 5) and computationally predicted (see Figure 3) matches, respectively. Mismatches are indicated by ‘x’. All proteins are aligned such that the C-terminal S motif aligns with the nucleotide at −4 with respect to the edited C (indicated in upper case).
(PDF)

Figure S2 Frequency of 6,1′ combinations in Arabidopsis PPR proteins. The most frequent combinations are shown (all those observed more than 30 times). Only tandem pairs of motifs (5362 in total) were considered in this analysis, where the first motif was either a P or S motif. Combinations observed in P motifs are shown in blue, those in S motifs in green.
(PDF)

Table S1 Alignments of PPR10 to the PPR10 RNA footprint ranked by p-value. The table shows the top 100 alignments out of the 29400 possible. The two alignments shaded in yellow correspond to the alignments depicted in Figure 2. Orientation: forward indicates N->C, 5′-3′; reverse indicates N->C, 3′-5′. Offset: distance from start of RNA sequence to first PPR motif. Gap position: nucleotide at which gap introduced between protein motifs. Gap length: length of gap in nucleotides. 17-mer: position (from 1 to 35) within the PPR motifs used to constitute the 17-mer sequence of amino acids used for the alignment. P-value: probability that amino acids and nucleotides are arranged independently of each other, as calculated by Fisher's Exact Test. None of the 29400 alignments exceed the threshold for significance at the 5% level if a threshold corrected for the total number of tests is used (5% threshold using the Šidák correction = 1.74E-06).
(PDF)

Table S2 Correlations between amino acids at specific positions within PPR motifs and aligned nucleotides. Contingency tables (amino acids *versus* nucleotides) were constructed from the alignments in Figure 2 and Figure S1. Each 20×4 table was tested for independent assortment of amino acids and nucleotides using a chi-squared test (after first removing any empty rows from the table). P-values from the tests are shown in the table, with those values that are significant for both P and S motifs highlighted (a 1% significance threshold was used, corrected for multiple tests using the Šidák correction). Rows: amino acid positions within the motifs. Columns: 0 indicates the motif aligned with the nucleotide, −1 the preceding motif, +1 the following motif.
(PDF)

Table S3 Correlations between amino acids at positions 6, 1′ and aligned nucleotides. The tables show frequencies of co-occurrence of amino acids and nucleotides from the alignments in Figure 2 and Figure S1. A. P motifs, positions 6, 1′ versus each nucleotide. B. S motifs, positions 6, 1′ versus each nucleotide. C. P motifs, position 6 versus purines (R), pyrimidines (Y). D. S motifs, position 6 versus purines (R), pyrimidines (Y). P-values were calculated using G-tests. P-values in A and B are for the most positively correlated nucleotide. Significance was evaluated at 5% allowing for multiple testing (using the Šidák correction). Green shading indicates significantly correlated, magenta shading indicates significantly anti-correlated.
(PDF)
