# Supplementary material for: A Combinatorial Amino Acid Code for RNA Recognition by Pentatricopeptide Repeat Proteins
Source: PLoS Genet. 2012 Aug 16;8(8):e1002910. doi: 10.1371/journal.pgen.1002910 (PMC3420917; doi:10.1371/journal.pgen.1002910)
Supplement: Figure S1 — Alignments of PPR editing factors to their target sites. For each factor, the name of the protein and its editing site are listed, then successively the types of PPR motif, the amino acids at position 6, the amino acids at position 1′, an indication of the degree to which these amino acids ‘match’ the RNA using the code developed in this work, and lastly the RNA sequence (in lower case). ‘:’ and ‘.’ indicate experimentally validated (see Figure 5) and computationally predicted (see Figure 3) matches, respectively. Mismatches are indicated by ‘x’. All proteins are aligned such that the C-terminal S motif aligns with the nucleotide at −4 with respect to the edited C (indicated in upper case). (PDF) [file pgen.1002910.s001.pdf]

```
CRR4 ndhD(117166) [1]
   LPPLSSSSSPLSPLS
   STNSNNNNANVVNV
   NDDDDNDDDDGSD
    :: :::: :  :
ugguguaucuugucuuuaccaC

CRR21 ndhD(116785) [2]
 PLSPLSPLSPLSPPPLSPLS
 FGTAPSNSTNASNNTTTNTG
 GNNDCNTDDDDDNNNLEDC
   x. .. :. .::: .:
caauguacagcggucaaauaggauC

CLB19 rpoA(78691) [3]
      PLSSPLSPLS
      TITNTINNTG
     TNHNDDNTDR
      : :.: x.
uguauuacacgugcaaaauC

CLB19 clpP(69942) [3]
      PLSSPLSPLS
      TITNTINNTG
     TNHNDDNTDR
      : :x: ..
agcaacagaagcccaagcuC

LPA66 psbF(63985) [4]
     SPLSSPLSPLS
     ANPSNNVSSIG
     NNDESNDNSR
     xx ..: .x
cuagcuguaccuaccguuuC

OTP80 rpl23 [5]
 SPLSSPLSPLS
 FTTLTTVNN
 NDSDDNSDN
  :  :: ::
augucgaaucaggauC

OTP81 rps12(69553) [5]
   SPLSPLSSPLSPLS
   SNPNNVNTNVSS
   NNDDSNDDNNDN
   .x :. :x: ..
ggaaauucuuuuugaaauauC

OTP84 ndhB(94999) [5]
  PLSPLSPLSPLSPLSPLS
  IPNNVNNSSNANNMSNIA
 SDSNSNDDNKNDDNDNNS
    x. x: .: :: .:
augugugauagcaucuacuauacC

OTP84 ndhF(112349) [5]
  PLSPLSPLSPLSPLSPLS
  IPNNVNNSSNANNMSNIA
 SDSNSNDDNKNDDNDNNS
    :. .: .: :: .:
cgauccacuuacuucuauuauguC

OTP84 psbZ(35800) [5]
  PLSPLSPLSPLSPLSPLS
  IPNNVNNSSNANNMSNIA
 SDSNSNDDNKNDDNDNNS
    x. :: .: x: .:
uuuugcauuaauuauuacuucauC

OTP85 ndhD(116494) [5]
  SPLSPLSPLS
  ANPPNLTS
  DDNCNYDD
  x:  : :x
cuuuugccgucaaauC
```

```
OTP82 ndhB(95644) [6]
   PLSSPLSPLSPLS
   NPTTNVNNLTNVG
   NDDDDNDNASDK
   x .:. x: x.
aaaguagcugcuucagcuuC

OTP82 ndhG [6]
   PLSSPLSPLSPLS
   NPTTNVNNLTNVG
   NDDDDNDNASDK
   : .:: :: x:
cuuuuaguuuuucugggauC

CRR28 ndhB(96998) [7]
  SPLSPLSPLS
  GN.NN.NN
  SD.SD.DN
  .: .: x:
cuaucuuuguagcucC

CRR28 ndhD(116281) [7]
  SPLSPLSPLS
  GN.NN.NN
  SD.SD.DN
  .: .x x:
uaauuuaugcagcuuC

CRR22 ndhB(96419) [7]
 SPLSPLSPLSPLSPLS
 THTTNVTNMNNVTNLG
 LVDDSLNTNTDNHNS
 .x x. x. .: .:
caagcuuucccuagccccuuC

CRR22 ndhD(116281) [7]
 SPLSPLSPLSPLSPLS
 THTTNVTNMNNVTNLG
 LVDDSLNTNTDNHNS
 xx :. .. .. .:
uuaugcagcuucaacaucucC

CRR22 rpoB(25779) [7]
 SPLSPLSPLSPLSPLS
 THTTNVTNMNNVTNLG
 LVDDSLNTNTDNHNS
 .. x. :. .: .:
aauaucuauucuaguucuauC

RARE1 accD(57868) [8]
   PLSPLSPLSPLSPLS
   NQNTTTTSTSTSTID
   SSNPNKDENNSDNT
   . x. .: :x .:
guucagaaagaaucgagcuuuC

MEF11 cox3(218701) [9,10]
   LSPLSPLSPLSPLS
   GNTPCNCNCSSNVS
   PNNDNNDNSTNNG
    x:  : :  ::
ucuuaauacccCuauucuccC

MEF11 nad4(161816) [9,10]
   LSPLSPLSPLSPLS
   GNTPCNCNCSSNVS
   PNNDNNDNSTNNG
    x:  : x  :x
gacCgauacgauuaauugguC

MEF11 ccb203(257133) [9,10]
   LSPLSPLSPLSPLS
   GNTPCNCNCSSNVS
   PNNDNNDNSTNNG
    x:  : x  ::
ucuuaauauugugacuuuucC
```

```
MEF1 rps4(82161) [11]
  LSPLSPLSPLS
  CNSPSSSSNLA
  ISDDNNSNNT
   .x .x xx
cuaccuacucauuauuC

MEF1 nad7(137931) [11]
  LSPLSPLSPLS
  CNSPSSSSNLA
  ISDDNNSNNT
   .. .. .:
auacagaagguuuuucC

MEF1 nad2(329886) [11]
  LSPLSPLSPLS
  CNSPSSSSNLA
  ISDDNNSNNT
   xx .. x:
aaaacgaauccuauuuC

YS1 rpoB(25992) [12]
 PLSPLSPLSSPLSPLS
 NPNTVSNLNNNMTTVG
 DDNNNNDWTNDDDDE
 x x: x: .:: ::
aaugaauucccuuggaacuuC

REME1 nad2-558 [13]
    LSPLSPLSPLS
    SNTSSNTNSVG
    MNNDDEDDNV
     :: .. ..
gaucuuaggugcauuuucC

REME1 orfX-552 [13]
    LSPLSPLSPLS
    SNTSSNTNSVG
    MNNDDEDDNV
     :: xx :.
cauuccaucgguaugcucC

SLO1 nad4(162141) [14]
   PLSPLSSPLSPLS
   NPNNINTNITTIS
   DVDDTTDDNNDQ
   : :. .:x ::
guuuuucccgaaagcgugcC

SLO1 nad9(23908) [14]
   PLSPLSSPLSPLS
   NPNNINTNITTIS
   DVDDTTDDNNDQ
   x :: ..: ::
ucuauuuccaucagccggcC

MEF18 nad4(167599) [15]
  LSSSLSSPLSSPLSPLS
  VNNTATNGVANNVANVG
 TNDSNRNDSYHDDNTND
   ..x ::. .:: x:
uuccuccauaaaauucuccgauuC

MEF19 ccb206(31028) [15]
    SPLSPLSPLSPLS
    SNPTGVTNLCTLG
    SDNNNNTDDDDT
    x: xx .:  :
ucuuuCauCgauugguuauuC

MEF21 cox3(218536) [15]
    SPLSSPLSPLS
    TNPNNTINSLG
    NDHDTDQDNK
    :. :.: :.
uaggacCucgauaugguuC
```

```
MEF22 nad3(260858) [15]
   SPLSPLSPLSPLS
   TNPNTVINTSSLA
   QDDTDENDDDNQ
   xx x:  : ..
gaaugugguuucgauccuuC

MEF9 nad7(133233) [16]
  SSSPLSSSSPLSPLS
  NNNSANNNNNTNNVS
  DDNLLNDDHDDESQ
  :::. x.:.: ..
ucuucaagcuuuaccuuauuC

MEF14 matR-1895 [17]
    PLSPLSPLSPLS
    NPSTVNNSTTVG
    TDNDGDNDNNG
    x x. :: ::
cucaaguacuccaaagacuC

OTP87 atp1(302512) [18]
SPLSPLSPLSPLSPLSPLSPLS
KNGSNSTTTANCSAASSSSTVV
DNYNDDSNDRDDDDGDDEDDE
 : .: .: xx .x x. .:
gcuaugaaacaaguaugcgguaguuC

OTP87 nad7(132094) [18]
SPLSPLSPLSPLSPLSPLSPLS
KNGSNSTTTANCSAASSSSTVV
DNYNDDSNDRDDDDGDDEDDE
 : xx x. x: .. .. .:
ccaugacgacuaggaaaaggcaaauC

MEF7 ccb206-28 [19]
 SPLSPLSPLSPLSPLSPLSPLS
 NAVNNGSNLNNINNSNNASNVS
DNNDNTDNSMDGNDNEDDDNDR
 x. x. .. :. :: :x ..
ugagacgacuuuuucuugaacuauauC

MEF7 cob-325 [19]
 SPLSPLSPLSPLSPLSPLSPLS
 NAVNNGSNLNNINNSNNASNVS
DNNDNTDNSMDGNDNEDDDNDR
 :x :. xx :. :: :x x.
accuucauauuuuucgugguc uauauC

MEF7 nad2-1433 [19]
 SPLSPLSPLSPLSPLSPLSPLS
 NAVNNGSNLNNINNSNNASNVS
DNNDNTDNSMDGNDNEDDDNDR
 xx x. .. :. :: :: .:
agcaaugacuuccuuuuucauuacuuC

MEF7 nad4L-41 [19]
 SPLSPLSPLSPLSPLSPLSPLS
 NAVNNGSNLNNINNSNNASNVS
DNNDNTDNSMDGNDNEDDDNDR
 :. :. .. :. :: x: .:
auauuucacauuuucuaugauuauuuC

SLG1 nad3(260757) [20]
  LSPLSPLSPLSPLSPLSPLS
  LKNANNANNINNLNNMGTTA
 DCDSDDNHDDDDDETNDDDT
   x. :: .: :. .: .:
aaccuuuuucuuuccuugggcaguaC

MEF3 atp4-89 [21]
    SPLSPLSPLSPLS
    SNVPGIATATSVA
   ENDDNDSNNDNNE
    .x  x .: :.
auuaaguucgaagaagaucuC
```

```
PPR2263 nad5-1550 [22]
  SPLSPLSPLSPLSPLS
  TNASNATNVTNSTNLT
 DDDDDDHDNDTNNNTG
  x: .. :: x: :.
aguuugcugcuccaaccauuaC

MEF29 nad5-1550 [22]
  SPLSPLSPLSPLSPLS
  TNASNLTNVTNTTNLA
 DDNEDDHDRHSNSNTS
  x: .. :. X: :.
aguuugcugcuccaaccauuaC

PPR2263 cob-908 [22]
  SPLSPLSPLSPLSPLS
  TNASNATNVTNSTNLT
 DDDDDDHDNDTNNNTG
  :x .: :: .: :.
gggguguagccgcaauagcacC

MEF29 cob-908 [22]
  SPLSPLSPLSPLSPLS
  TNASNLTNVTNTTNLA
 DDNEDDHDRHSNSNTS
  :x .: :. .: :.
gggguguagccgcaauagcacC

PpPPR_56 nad3 [23]
   LSPLSPLSPLSPLS
   VNNMTTVTNLTNVN
  DNSNDDNDDDDNDD
    .: :: :: ::
ugauuuggaagucaccuuuuC

PpPPR_56 nad4 [23]
   LSPLSPLSPLSPLS
   VNNMTTVTNLTNVN
  DNSNDDNDDDDNDD
    x: x. :x ::
ucgaauucgugggacuauugC

PpPPR_77 cox2 [23]
 LSPLSPLSPLSPLSPLSPLS
 VNNINNITNLNNLNNANTTG
DDDNDDDDDNDDGNDDDDDT
  .: :. .x .. :: x.
uacuaucaaagcuauuggacaucaaC

PpPPR_77 cox3 [23]
 LSPLSPLSPLSPLSPLSPLS
 VNNINNITNLNNLNNANTTG
DDDNDDDDDNDDGNDDDDDT
  x: :: .x .x .: .x
uggcuuugaagcagcugcuugguacC

PpPPR_91 nad5-2 [23]
  LSPLSPLSPLSSPLSPLS
  GNSLTTVNSMTTNITNTG
 NDDNDNNDDNDNNDDDNR
   .. :: :. .:: ::
cugcacaaauaggauugcauacuC

PpPPR_71 ccmF-2 [24]
   LSPLSPLSPLSPLSPLS
   VNNLTNLTTMTSINNVG
  DNTSDDNDDDDDDHDNT
    .. .: :: :x .:
uguucccacaggugcuccuuuuuC

PpPPR_78 rps14 [25]
 LSPLSPLSPLSPLSPLSPLS
 NNMMNTITNVNTTNNINNGV
DTHDDSNDDNDDNDDDDNTN
  .  .: :x :: :: :.
uacguuaugaauauuuuuuaaaguC
```

```
PpPPR_78 cox1 [25]
 LSPLSPLSPLSPLSPLSPLS
 NNMMNTITNVNTTNNINNGV
DTHDDSNDDNDDNDDDDNTN
  .  x: :: :: x: :.
auccugaggucuauauucuaauuuC

PpPPR_79 nad5-1 [25]
    LSPLSPLSPLSPLSPLS
    VNNVNTMTNTNTMNNVA
   DNSDDDSDDDDDNDDNT
     .: :x :x :: ::
guagacuuuucgacuauuuuugcuC

OGR1 cox2-C167 [26]
    LSPLSPLS
    STNVNNLG
   DDDNNTDN
     :: .:
uuuugguuuucguauC

OGR1 cox3-C572 [26]
    LSPLSPLS
    STNVNNLG
   DDDNNTDN
     :: .:
uacuggcucuaguauC

OGR1 ccmC-C458 [26]
    LSPLSPLS
    STNVNNLG
   DDDNNTDN
     :: ..
aacuggcuucuauuuC

OGR1 nad2-C1457 [26]
    LSPLSPLS
    STNVNNLG
   DDDNNTDN
     :: .:
aaauggcacucaguuC

OGR1 nad4-C401 [26]
    LSPLSPLS
    STNVNNLG
   DDDNNTDN
     .: X.
uaaugaucgccguguC

OGR1 nad4-C416 [26]
    LSPLSPLS
    STNVNNLG
   DDDNNTDN
     .: .:
ucugcaugcuggaucC

OGR1 nad4-C433 [26]
    LSPLSPLS
    STNVNNLG
   DDDNNTDN
     .: .:
cuacuauucuauguuC
```

Figure S1
Alignments of PPR editing factors to their target sites. For each factor, the name of the protein and its editing site are listed, then successively the types of PPR motif, the amino acids at position 6, the amino acids at position 1', an indication of the degree to which these amino acids 'match' the RNA using the code developed in this work, and lastly the RNA sequence (in lower case). ':' and '.' indicate experimentally validated (see Figure 4) and computationally predicted (see Figure 3) matches, respectively. Mismatches are indicated by 'x'. All proteins are aligned such that the C-terminal S motif aligns with the nucleotide at -4 with respect to the edited C (indicated in upper case).

References

1. Kotera E, Tasaka M, Shikanai T. 2005. A pentatricopeptide repeat protein is essential for RNA editing in chloroplasts. Nature 433(7023): 326-330.
2. Okuda K, Myouga F, Motohashi R, Shinozaki K, Shikanai T. 2007. Conserved domain structure of pentatricopeptide repeat proteins involved in chloroplast RNA editing. Proc Natl Acad Sci U S A 104(19): 8178-8183.
3. Chateigner-Boutin AL, Ramos-Vega M, Guevara-Garcia A, Andres C, de la Luz Gutierrez-Nava M, Cantero A, Delannoy E, Jimenez LF, Lurin C, Small I, Leon P. 2008. CLB19, a pentatricopeptide repeat protein required for editing of *rpoA* and clpP chloroplast transcripts. Plant J 56(4): 590-602.
4. Cai W, Ji D, Peng L, Guo J, Ma J, Zou M, Lu C, Zhang L. 2009. LPA66 is required for editing psbF chloroplast transcripts in Arabidopsis. Plant Physiol 150(3): 1260-1271.
5. Hammani K, Okuda K, Tanz SK, Chateigner-Boutin AL, Shikanai T, Small I. 2009. A study of new Arabidopsis chloroplast RNA editing mutants reveals general features of editing factors and their target sites. Plant Cell 21(11): 3686-3699.
6. Okuda K, Hammani K, Tanz SK, Peng L, Fukao Y, Myouga F, Motohashi R, Shinozaki K, Small I, Shikanai T. 2010. The pentatricopeptide repeat protein OTP82 is required for RNA editing of plastid *ndhB* and *ndhG* transcripts. Plant J 61(2): 339-49
7. Okuda K, Chateigner-Boutin AL, Nakamura T, Delannoy E, Sugita M, Myouga F, Motohashi R, Shinozaki K, Small I, Shikanai T. 2009. Pentatricopeptide repeat proteins with the DYW motif have distinct molecular functions in RNA editing and RNA cleavage in Arabidopsis chloroplasts. Plant Cell 21(1): 146-156.
8. Robbins JC, Heller WP, Hanson MR. 2009. A comparative genomics approach identifies a PPR-DYW protein that is essential for C-to-U editing of the Arabidopsis chloroplast *accD* transcript. RNA 15(6): 1142-1153.
9. Tang J, Kobayashi K, Suzuki M, Matsumoto S, Muranaka T. 2010. The mitochondrial PPR protein LOVASTATIN INSENSITIVE 1 plays regulatory roles in cytosolic and plastidial isoprenoid biosynthesis through RNA editing. Plant J 61(3): 456-466.
10. Verbitskiy D, Zehrmann A, van der Merwe JA, Brennicke A, Takenaka M. 2010. The PPR protein encoded by the LOVASTATIN INSENSITIVE 1 gene is involved in RNA editing at three sites in mitochondria of *Arabidopsis thaliana*. Plant J 61(3): 446-455.
11. Zehrmann A, Verbitskiy D, van der Merwe JA, Brennicke A, Takenaka M. 2009. A DYW domain-containing pentatricopeptide repeat protein is required for RNA editing at multiple sites in mitochondria of *Arabidopsis thaliana*. Plant Cell 21(2): 558-567.
12. Zhou W, Cheng Y, Yap A, Chateigner-Boutin AL, Delannoy E, Hammani K, Small I, Huang J. 2008. The Arabidopsis gene *YS1* encoding a DYW protein is required for editing of *rpoB* transcripts and the rapid development of chloroplasts during early growth. Plant J 58(1):82-96.
13. Bentolila S, Knight W, Hanson M. 2010. Natural variation in Arabidopsis leads to the identification of REME1, a pentatricopeptide repeat-DYW protein controlling the editing of mitochondrial transcripts. Plant Physiol 154(4): 1966-1982.
14. Sung TY, Tseng CC, Hsieh MH. 2010. The SLO1 PPR protein is required for RNA editing at multiple sites with similar upstream sequences in Arabidopsis mitochondria. Plant J 63(3): 499-511.
15. Takenaka M, Verbitskiy D, Zehrmann A, Brennicke A. 2010. Reverse genetic screening identifies five E-class PPR proteins involved in RNA editing in mitochondria of Arabidopsis thaliana. J Biol Chem 285(35): 27122-27129.

16. Takenaka M. 2010. MEF9, an E-subclass pentatricopeptide repeat protein, is required for an RNA editing event in the *nad7* transcript in mitochondria of Arabidopsis. Plant Physiol 152(2): 939-947.
17. Verbitskiy D, Härtel B, Zehrmann A, Brennicke A, Takenaka M. 2011. The DYW-E-PPR protein MEF14 is required for RNA editing at site matR-1895 in mitochondria of *Arabidopsis thaliana*. FEBS Lett. 585(4): 700-4.
18. Hammani K, des Francs-Small CC, Takenaka M, Tanz SK, Okuda K, Shikanai T, Brennicke A, Small I. 2011. The pentatricopeptide repeat protein OTP87 is essential for RNA editing of *nad7* and *atp1* transcripts in Arabidopsis mitochondria. J Biol Chem 286(24): 21361-71.
19. Zehrmann A, van der Merwe J, Verbitskiy D, Härtel B, Brennicke A, Takenaka M. 2012. The DYW-class PPR protein MEF7 is required for RNA editing at four sites in mitochondria of *Arabidopsis thaliana*. RNA Biol 9(2), in press
20. Yuan H, Liu D. 2012. Functional disruption of the pentatricopeptide protein SLG1 affects mitochondrial RNA editing, plant development, and responses to abiotic stresses in Arabidopsis. Plant J 70(3): 432-44.
21. Verbitskiy D, Merwe JA, Zehrmann A, Härtel B, Takenaka M. 2012. The E-class PPR protein MEF3 of *Arabidopsis thaliana* can also function in mitochondrial RNA editing with an additional DYW domain. Plant Cell Physiol 53(2): 358-67.
22. Sosso D, Mbelo S, Vernoud V, Gendrot G, Dedieu A, Chambrier P, Dauzat M, Heurtevin L, Guyon V, Takenaka M, Rogowsky PM. 2012. PPR2263, a DYW-Subgroup Pentatricopeptide Repeat Protein, Is Required for Mitochondrial *nad5* and *cob* Transcript Editing, Mitochondrion Biogenesis, and Maize Growth. Plant Cell 24(2): 676-91.
23. Ohtani S, Ichinose M, Tasaki E, Aoki Y, Komura Y, Sugita M. 2010. Targeted gene disruption identifies three PPR-DYW proteins involved in RNA editing for five editing sites of the moss mitochondrial transcripts. Plant Cell Physiol 51(11): 1942-1949.
24. Tasaki E, Hattori M, Sugita M. 2010. The moss pentatricopeptide repeat protein with a DYW domain is responsible for RNA editing of mitochondrial *ccmFc* transcript. Plant J 62(4): 560-70
25. Uchida M, Ohtani S, Ichinose M, Sugita C, Sugita M. 2011. The PPR-DYW proteins are required for RNA editing of *rps14*, *cox1* and *nad5* transcripts in *Physcomitrella patens* mitochondria. FEBS Lett 585(14): 2367-71.
26. Kim SR, Yang JI, Moon S, Ryu CH, An K, Kim KM, Yim J, An G. 2009. Rice OGR1 encodes a pentatricopeptide repeat-DYW protein and is essential for RNA editing in mitochondria. Plant J 59(5): 738-749.
