# Supplementary material for: A Combinatorial Amino Acid Code for RNA Recognition by Pentatricopeptide Repeat Proteins
Source: PLoS Genet. 2012 Aug 16;8(8):e1002910. doi: 10.1371/journal.pgen.1002910 (PMC3420917; doi:10.1371/journal.pgen.1002910)
Supplement: Figure S2 — Frequency of 6,1′ combinations in Arabidopsis PPR proteins. The most frequent combinations are shown (all those observed more than 30 times). Only tandem pairs of motifs (5362 in total) were considered in this analysis, where the first motif was either a P or S motif. Combinations observed in P motifs are shown in blue, those in S motifs in green. (PDF) [file pgen.1002910.s002.pdf]

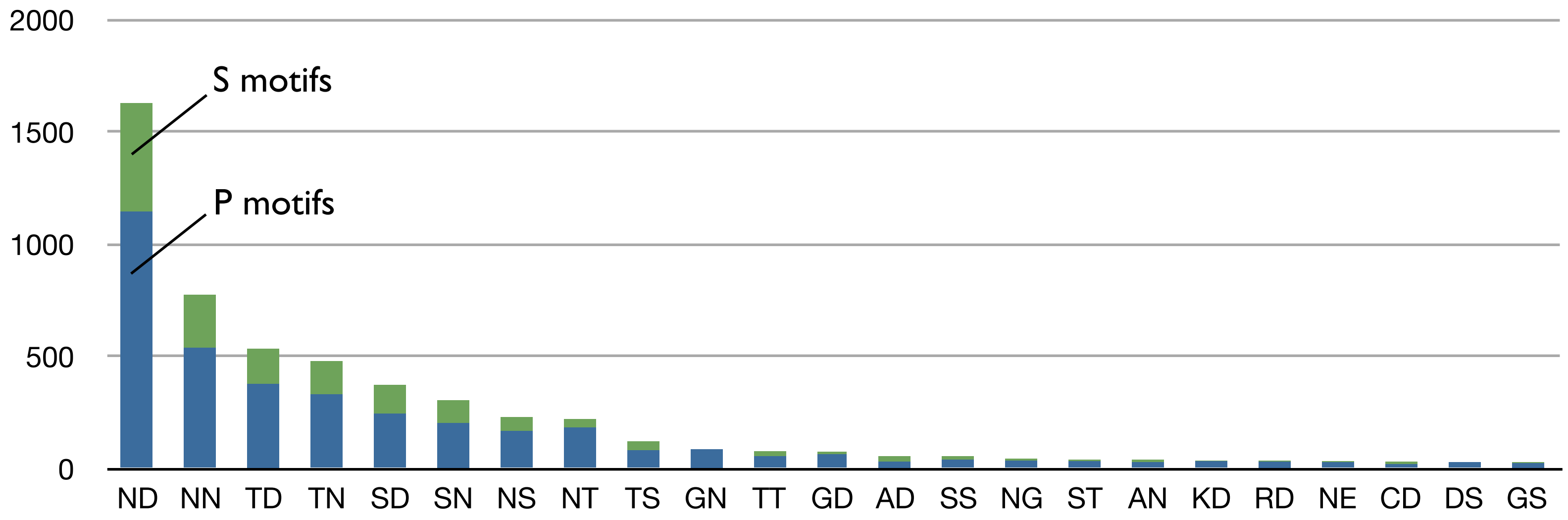

Figure S2. Frequency of 6,1' combinations in Arabidopsis PPR proteins. The most frequent combinations are shown (all those observed more than 30 times). Only tandem pairs of motifs (5362 in total) were considered in this analysis, where the first motif was either a P or S motif. Combinations observed in P motifs are shown in blue, those in S motifs in green.
