# Supplementary material for: A Combinatorial Amino Acid Code for RNA Recognition by Pentatricopeptide Repeat Proteins
Source: PLoS Genet. 2012 Aug 16;8(8):e1002910. doi: 10.1371/journal.pgen.1002910 (PMC3420917; doi:10.1371/journal.pgen.1002910)
Supplement: Table S1 — Alignments of PPR10 to the PPR10 RNA footprint ranked by p-value. The table shows the top 100 alignments out of the 29400 possible. The two alignments shaded in yellow correspond to the alignments depicted in Figure 2. Orientation: forward indicates N->C, 5′-3′; reverse indicates N->C, 3′-5′. Offset: distance from start of RNA sequence to first PPR motif. Gap position: nucleotide at which gap introduced between protein motifs. Gap length: length of gap in nucleotides. 17-mer: position (from 1 to 35) within the PPR motifs used to constitute the 17-mer sequence of amino acids used for the alignment. P-value: probability that amino acids and nucleotides are arranged independently of each other, as calculated by Fisher's Exact Test. None of the 29400 alignments exceed the threshold for significance at the 5% level if a threshold corrected for the total number of tests is used (5% threshold using the Šidák correction = 1.74E-06). (PDF) [file pgen.1002910.s003.pdf]

Table S1. Alignments of PPR10 to the PPR10 RNA footprint ranked by p-value.
The table shows the top 100 alignments out of the 29400 possible. The two alignments shaded in yellow correspond to the alignments depicted in Figure 2. Orientation: forward indicates N->C, 5'-3'; reverse indicates N->C, 3'-5'. Offset: distance from start of RNA sequence to first PPR motif. Gap position: nucleotide at which gap introduced between protein motifs. Gap length: length of gap in nucleotides. 17-mer: position (from 1 to 35) within the PPR motifs used to constitute the 17-mer sequence of amino acids used for the alignment. P-value: probability that amino acids and nucleotides are arranged independently of each other, as calculated by Fisher's Exact Test. None of the 29400 alignments exceed the threshold for significance at the 5% level if a threshold corrected for the total number of tests is used (5% threshold using the Šidák correction = 1.74E-06).

orientation	offset	gap position	gap length	17-mer	p-value
forward	2	8	2	1	0.0001631
forward	2	6	3	33	0.0006832
reverse	1	5	4	14	0.0008227
forward	3	13	1	25	0.001122
forward	3	14	1	25	0.001122
forward	3	15	1	25	0.001122
forward	2	6	2	1	0.001168
forward	1	2	3	8	0.001234
reverse	1	9	5	8	0.001234
reverse	1	10	5	8	0.001234
forward	3	6	1	2	0.001272
forward	3	7	1	2	0.001272
forward	2	10	2	1	0.001283
reverse	1	12	2	8	0.001508
reverse	1	10	4	8	0.001618
reverse	1	8	5	8	0.001848
reverse	1	13	2	8	0.001892
reverse	0	13	3	8	0.001892
forward	1	4	3	2	0.001988
forward	2	9	2	1	0.00202
forward	2	2	3	21	0.002101
forward	2	8	3	33	0.002143
reverse	0	12	3	13	0.002236
forward	4	2	1	21	0.002262
forward	4	5	1	21	0.002262
forward	4	6	1	21	0.002262
reverse	3	10	2	21	0.002262
forward	5	6	1	12	0.002342
forward	5	7	1	12	0.002342
reverse	2	9	3	16	0.002343
reverse	0	12	3	8	0.002345
reverse	0	7	2	30	0.002458
reverse	2	8	3	16	0.002618
reverse	1	6	4	14	0.002622
forward	2	5	2	1	0.002766
forward	3	3	3	3	0.002921
forward	3	4	3	3	0.002921
forward	3	15	2	25	0.002943
forward	3	14	3	25	0.002943
forward	3	15	3	25	0.002943
reverse	1	11	4	8	0.002962
reverse	3	7	2	21	0.003087

forward	4	7	2	23	0.003144
reverse	2	10	2	10	0.003156
forward	0	6	1	14	0.003686
reverse	0	7	3	17	0.003755
reverse	1	10	1	35	0.003959
reverse	1	11	1	35	0.003959
reverse	1	11	2	8	0.004072
forward	2	3	3	21	0.004105
reverse	0	10	5	8	0.004148
reverse	0	11	5	8	0.004148
forward	0	5	1	14	0.004178
forward	1	8	1	5	0.004202
forward	1	9	1	5	0.004202
reverse	3	8	3	15	0.004319
reverse	3	9	2	21	0.004363
forward	2	7	3	33	0.004477
forward	2	9	1	7	0.004548
forward	2	10	1	7	0.004548
forward	2	13	4	7	0.004548
reverse	0	14	5	25	0.004601
reverse	0	15	5	25	0.004601
reverse	3	8	2	21	0.004606
reverse	1	7	2	8	0.004628
reverse	0	7	3	8	0.004628
forward	3	2	1	8	0.004795
forward	2	2	2	8	0.004795
forward	2	3	2	8	0.004795
forward	1	3	3	8	0.004795
forward	3	8	2	6	0.004816
forward	2	5	1	28	0.005208
forward	2	6	1	28	0.005208
reverse	1	12	4	8	0.005389
reverse	1	13	4	8	0.005389
reverse	0	13	5	8	0.005389
forward	2	2	2	1	0.005535
forward	2	3	2	1	0.005535
forward	0	5	1	4	0.005624
forward	1	12	1	22	0.005638
forward	1	13	1	22	0.005638
forward	2	4	2	8	0.005768
reverse	0	12	5	8	0.005913
forward	3	5	3	3	0.006074
forward	3	10	1	2	0.006173
forward	3	11	1	2	0.006173
forward	5	8	1	12	0.00619
forward	5	9	1	12	0.00619
forward	1	12	1	8	0.006253
forward	1	13	1	8	0.006253
forward	1	6	2	28	0.006338
reverse	3	9	2	15	0.006486
reverse	0	11	3	13	0.006758
reverse	3	5	3	15	0.006787
reverse	3	6	3	15	0.006787
reverse	2	5	4	15	0.006787
reverse	0	15	1	8	0.007096
forward	2	14	1	7	0.00714

forward	2	15	1	7	0.00714
reverse	1	14	4	1	0.007143
