# Supplementary material for: A Combinatorial Amino Acid Code for RNA Recognition by Pentatricopeptide Repeat Proteins
Source: PLoS Genet. 2012 Aug 16;8(8):e1002910. doi: 10.1371/journal.pgen.1002910 (PMC3420917; doi:10.1371/journal.pgen.1002910)
Supplement: Table S2 — Correlations between amino acids at specific positions within PPR motifs and aligned nucleotides. Contingency tables (amino acids versus nucleotides) were constructed from the alignments in Figure 2 and Figure S1. Each 20×4 table was tested for independent assortment of amino acids and nucleotides using a chi-squared test (after first removing any empty rows from the table). P-values from the tests are shown in the table, with those values that are significant for both P and S motifs highlighted (a 1% significance threshold was used, corrected for multiple tests using the Šidák correction). Rows: amino acid positions within the motifs. Columns: 0 indicates the motif aligned with the nucleotide, −1 the preceding motif, +1 the following motif. (PDF) [file pgen.1002910.s004.pdf]

Table S2. Correlations between amino acids at specific positions within PPR motifs and aligned nucleotides. Contingency tables (amino acids *versus* nucleotides) were constructed from the alignments in Figure 2 and Figure S1. Each 20 x 4 table was tested for independent assortment of amino acids and nucleotides using a chi-squared test (after first removing any empty rows from the table). P-values from the tests are shown in the table, with those values that are significant for both P and S motifs highlighted (a 1% significance threshold was used, corrected for multiple tests using the Šidák correction). Rows: amino acid positions within the motifs. Columns: 0 indicates the motif aligned with the nucleotide, -1 the preceding motif, +1 the following motif.

	P				S		
	-1	0	+1		-1	0	+1
1	1.68E-02	5.25E-01	5.02E-08		1.74E-02	1.43E-01	1.43E-10
2	1.22E-01	6.93E-02	3.68E-02		3.49E-04	4.67E-01	9.23E-02
3	2.63E-01	9.30E-03	8.42E-04		4.08E-04	1.01E-05	5.24E-02
4	1.92E-01	1.25E-01	4.03E-07		2.51E-04	1.11E-01	2.15E-07
5	7.14E-05	5.68E-01	1.19E-01		2.29E-02	2.09E-03	6.16E-02
6	3.93E-01	6.93E-28	5.59E-01		1.34E-02	5.75E-12	1.70E-01
7	2.69E-04	9.65E-02	1.89E-02		1.67E-01	5.27E-01	2.03E-02
8	1.80E-02	1.00E-01	2.22E-01		2.03E-03	6.77E-03	1.99E-02
9	2.50E-01	3.85E-03	8.12E-02		1.82E-04	3.06E-03	5.39E-02
10	9.14E-04	4.55E-02	1.01E-02		3.55E-01	1.93E-03	1.58E-01
11	2.24E-01	6.35E-02	4.60E-01		3.49E-02	7.75E-02	4.42E-02
12	3.47E-03	8.28E-03	6.21E-01		3.14E-04	3.33E-01	1.65E-01
13	2.32E-01	3.45E-02	2.80E-01		5.34E-02	3.32E-01	3.20E-04
14	5.42E-02	4.59E-03	7.16E-02		1.98E-03	8.92E-02	1.40E-03
15	3.41E-02	2.59E-03	1.98E-01		8.24E-01	6.76E-01	7.67E-03
16	4.27E-02	6.01E-01	2.06E-01		4.77E-01	2.91E-01	3.84E-01
17	1.94E-02	1.72E-01	9.16E-01		5.52E-02	1.03E-01	4.35E-02
18	8.29E-03	1.17E-01	9.39E-02		5.12E-01	2.46E-01	2.16E-01
19	1.61E-01	5.41E-01	4.40E-03		3.95E-02	1.72E-01	1.43E-03
20	1.57E-01	1.78E-01	1.51E-01		2.18E-04	3.74E-03	1.07E-01
21	2.26E-02	1.22E-01	8.41E-04		2.55E-02	6.81E-02	4.50E-02
22	7.73E-02	1.73E-01	1.24E-01		7.52E-02	7.30E-01	1.85E-01
23	6.00E-03	2.83E-03	3.29E-01		3.60E-01	1.60E-01	2.05E-03
24	4.57E-01	2.07E-04	3.53E-01		5.97E-04	1.71E-01	2.80E-01
25	5.61E-01	1.20E-02	1.27E-01		1.42E-01	1.56E-01	2.85E-02
26	1.27E-02	1.60E-05	8.81E-02		4.57E-03	5.87E-02	3.60E-02
27	2.26E-02	7.69E-02	3.82E-02		6.66E-03	8.42E-03	7.56E-02
28	6.61E-02	4.08E-02	4.06E-03		3.62E-01	2.46E-01	1.60E-01
29	3.88E-02	1.62E-02	6.03E-02		1.59E-02	8.14E-01	3.77E-02
30	2.07E-01	7.95E-03	4.96E-01		1.51E-02	6.21E-02	9.96E-02
31	1.00E-01	4.61E-02	1.10E-02		1.04E-01	2.74E-01	1.48E-01
32	2.83E-01	2.14E-01	3.58E-03				
33	5.85E-01	1.88E-02	1.07E-01		1% significance threshold:		1.08E-04

	P				S		
	-1	0	+1		-1	0	+1
**34**	7.49E-03	3.72E-01	3.56E-02				
**35**	8.29E-02	5.43E-01	1.61E-03				
	1% significance threshold:		9.57E-05				
