# Supplementary material for: A Combinatorial Amino Acid Code for RNA Recognition by Pentatricopeptide Repeat Proteins
Source: PLoS Genet. 2012 Aug 16;8(8):e1002910. doi: 10.1371/journal.pgen.1002910 (PMC3420917; doi:10.1371/journal.pgen.1002910)
Supplement: Table S3 — Correlations between amino acids at positions 6, 1′ and aligned nucleotides. The tables show frequencies of co-occurrence of amino acids and nucleotides from the alignments in Figure 2 and Figure S1. A. P motifs, positions 6, 1′ versus each nucleotide. B. S motifs, positions 6, 1′ versus each nucleotide. C. P motifs, position 6 versus purines (R), pyrimidines (Y). D. S motifs, position 6 versus purines (R), pyrimidines (Y). P-values were calculated using G-tests. P-values in A and B are for the most positively correlated nucleotide. Significance was evaluated at 5% allowing for multiple testing (using the Šidák correction). Green shading indicates significantly correlated, magenta shading indicates significantly anti-correlated. (PDF) [file pgen.1002910.s005.pdf]

P motifs

A

6	1'	A	C	G	U	p-value
T	D	2	0	23	1	9.70E-18
T	N	23	0	2	0	1.34E-14
N	D	7	22	7	68	2.09E-09
N	N	6	46	3	31	2.39E-06
S	N	10	1	0	1	3.42E-06
N	S	1	12	0	3	1.50E-04
T	T	0	0	3	0	5.59E-04
S	S	5	1	0	0	1.02E-03
S	R	0	0	2	0	4.84E-03
S	C	2	0	0	0	1.21E-02
R	D	0	0	0	3	1.38E-02
T	S	3	0	0	1	2.01E-02
N	R	0	2	0	0	2.63E-02
A	D	0	0	2	1	3.56E-02
G	D	0	1	2	0	3.56E-02
H	M	0	0	0	2	4.43E-02
H	S	0	0	0	2	4.43E-02
N	C	0	5	0	3	5.06E-02
F	G	1	0	0	0	7.62E-02
G	H	1	0	0	0	7.62E-02
G	S	1	0	0	0	7.62E-02
S	G	1	0	0	0	7.62E-02
S	L	1	0	0	0	7.62E-02
T	P	1	0	0	0	7.62E-02
H	V	2	0	0	1	8.66E-02
G	N	0	1	0	0	1.16E-01
N	T	1	8	0	8	1.19E-01
N	G	1	2	0	5	1.35E-01
A	N	2	2	0	0	1.96E-01
M	D	0	1	1	0	2.22E-01
N	E	0	0	1	1	2.22E-01
T	R	1	0	1	0	2.22E-01
S	D	4	3	3	1	2.34E-01
C	S	0	1	0	2	2.88E-01
I	D	1	0	1	1	3.89E-01

B

6	R	Y	p-value
T	59	2	1.16E-25
N	27	216	4.10E-17
S	28	7	3.36E-08
G	4	2	1.09E-01
C	0	3	1.11E-01
R	0	3	1.11E-01
F	1	0	1.45E-01
A	4	3	2.22E-01
I	2	1	2.57E-01
M	1	1	6.53E-01
H	2	5	7.38E-01

S motifs

C

6	1'	A	C	G	U	p-value
T	D	9	2	27	4	4.72E-14
S	D	1	1	13	1	1.16E-09
S	N	20	2	1	5	8.69E-09
T	N	18	1	2	3	1.25E-08
N	D	5	13	5	44	1.20E-06
C	N	3	0	0	0	2.13E-03
N	T	0	12	3	4	2.14E-03
N	N	5	8	9	5	9.80E-03
G	S	2	0	0	0	1.21E-02
A	R	0	2	0	0	2.63E-02
T	H	1	0	2	0	3.56E-02
C	D	0	0	1	0	4.64E-02
G	D	0	0	1	0	4.64E-02
P	C	0	0	1	0	4.64E-02
A	H	1	0	0	0	7.62E-02
S	E	1	0	0	0	7.62E-02
T	E	1	0	0	0	7.62E-02
T	K	1	0	0	0	7.62E-02
S	K	2	0	1	0	8.66E-02
T	L	2	0	0	1	8.66E-02
L	D	0	1	0	0	1.16E-01
N	E	0	1	0	0	1.16E-01
A	T	0	0	0	1	1.55E-01
F	N	0	0	0	1	1.55E-01
I	N	0	0	0	1	1.55E-01
P	N	0	0	0	1	1.55E-01
S	S	0	0	0	1	1.55E-01
T	Q	0	0	0	1	1.55E-01
V	S	0	0	0	1	1.55E-01
K	D	0	2	1	0	1.79E-01
N	H	0	2	0	1	1.79E-01
A	D	0	0	1	1	2.22E-01
T	S	1	3	1	1	2.83E-01
A	N	1	0	0	1	3.60E-01
T	T	1	1	1	0	3.89E-01
N	S	2	4	1	5	7.06E-01

D

6	R	Y	p-value
T	67	17	2.72E-16
S	39	10	4.24E-10
N	30	99	4.32E-08
C	4	0	4.55E-02
A	3	5	8.33E-02
G	3	0	8.33E-02
K	1	2	3.17E-01
P	1	1	3.17E-01
F	0	1	1.00E+00
I	0	1	1.00E+00
L	0	1	1.00E+00
V	0	1	1.00E+00

Table S3. Correlations between amino acids at positions 6, 1' and aligned nucleotides. The tables show frequencies of co-occurrence of amino acids and nucleotides from the alignments in Fig. 2 and Fig. S1. **A**: P motifs, positions 6, 1' versus each nucleotide; **B**: S motifs, positions 6, 1' versus each nucleotide; **C**: P motifs, position 6 versus purines (R), pyrimidines (Y); **D**: S motifs, position 6 versus purines (R), pyrimidines (Y). P-values were calculated using G-tests. P-values in **A** and **B** are for the most positively correlated nucleotide. Significance was evaluated at 5% allowing for multiple testing (using the Šidák correction). Green shading indicates significantly correlated, magenta shading indicates significantly anti-correlated.
